# Association between sleep inertia and cognitive performance among older adults in the Wisconsin Sleep Cohort study

**DOI:** 10.1007/s44470-026-00133-4

**Published:** 2026-07-06

**Authors:** Jessica J. Love, Jesse D. Cook, Erika W. Hagen, Amanda Rasmunon, Laurel A. Ravelo, Mari Palta, Paul E. Peppard, David T. Plante

**Affiliations:** 1 Department of Psychiatry, School of Medicine and Public Health, University of Wisconsin–Madison, Madison, WI USA; 2 Department of Population Health Sciences, School of Medicine and Public Health, University of Wisconsin–Madison, Madison, WI USA; 3 Department of Psychology, University of Wisconsin–Madison, Madison, WI USA

**Keywords:** Sleep inertia, Older adults, Neurocognition, Hypersomnolence, Cognitive performance, Cognitive health

## Abstract

**Purpose:**

Since sleep inertia and hypersomnolence, broadly, have not been adequately studied in the context of neurocognitive performance, this investigation examined associations between sleep inertia severity, other measures of hypersomnolence, and outcomes from a standardized, cognitive testing battery in a well-characterized community-based sample of older adults.

**Methods:**

Wisconsin Sleep Cohort participants completed the Sleep Inertia Questionnaire (SIQ), Epworth Sleepiness Scale (ESS), Hypersomnia Severity Index (HSI) and six widely used cognitive tests. Linear regression examined relationships between hypersomnolence measures (SIQ, ESS, and HSI) and cognitive outcomes. Secondary analyses assessed relationships between SIQ subscales (physiological, cognitive, emotional, and responses) and cognitive outcomes. Adjusted models included covariates capturing demographic, psychosocial, sleep, and testing characteristics.

**Results:**

The sample (*N* = 461) was predominantly non-Hispanic white older adults (average age = 73.84 ± 6.66 years), with majority being male (55.8%). In unadjusted analyses, SIQ total score was associated with Grooved Pegboard (GPB), Trail Making Test-Part B (TMT-B), and Symbol Digits Modalities Test, while neither ESS nor HSI was associated with cognitive outcomes. Significant associations between SIQ and GPB and TMT-B remained in fully adjusted analyses. Physiological, cognitive, and emotional SIQ subscales were also associated with cognitive tests, particularly GPB and TMT-B.

**Conclusions:**

This study suggests that sleep inertia may be an important, specific symptom that is associated with cognitive performance in older adults. Future research is indicated to clarify the role of sleep inertia in aging, while exploring its potential value as a risk factor for cognitive impairment and target for intervention to support cognitive health.

**Brief summary:**

**Current Study/Study Rationale:**

Sleep inertia is a common sleep-related symptom, yet it remains insufficiently studied, particularly regarding neurocognitive performance. The Sleep Inertia Questionnaire (SIQ) is a validated measure of sleep inertia, but has not been studied in older populations at risk for cognitive impairment. Clarifying relationships between SIQ scores and neurocognitive outcomes may identify those at greater risk for cognitive impairment.

**Study Impact:**

Sleep inertia is a common sleep-related symptom, yet it remains insufficiently studied, particularly regarding neurocognitive performance. The Sleep Inertia Questionnaire (SIQ) is a validated measure of sleep inertia, but has not been studied in older populations at risk for cognitive impairment. Clarifying relationships between SIQ scores and neurocognitive outcomes may identify those at greater risk for cognitive impairment.

**Supplementary information:**

The online version contains supplementary material available at 10.1007/s44470-026-00133-4.

## Background

Sleep health is established as a key contributing factor to neurocognitive performance as well as long-term cognitive health [1, 2]. Previous research has clarified that acute and chronic sleep deficiencies meaningfully impair many domains of cognitive function [3, 4]. Additionally, highly prevalent sleep problems, such as insomnia and obstructive sleep apnea, are associated with impaired cognition and heightened risk for neurocognitive disorders [5–7]. Cognitive deficiencies are also common among neurological disorders of excessive daytime sleepiness (EDS) such as narcolepsy [8]. A recent meta-analysis demonstrated impaired attention, executive function, and memory are associated with central disorders of hypersomnolence, with secondary analyses implicating sustained attention as the domain most commonly impaired in these disorders [9].

Sleep inertia is a specific symptom associated with disorders of hypersomnolence for which associations with daytime neurocognitive batteries have not been previously examined. Sleep inertia is defined as the period of cognitive, physical, and/or psychological impairment during the transition from sleep to wakefulness [10]. Although sleep inertia is a near universal experience with symptoms generally dissipating within 30 min upon awakening [10], the duration and intensity of sleep inertia can vary drastically between individuals [11]. Notably, prolonged, debilitating sleep inertia is a distinguishing feature of idiopathic hypersomnia [12, 13], a disorder defined by hypersomnolence that presents without a clear underlying explanatory condition. Problems with sleep inertia can occur in other sleep disorders such as obstructive sleep apnea [14], insufficient sleep syndrome [10], and circadian rhythm sleep–wake disorders [15]. Additionally, sleep inertia is common in psychiatric conditions [11], particularly mood disorders [16], which are frequently associated with cognitive deficits [17, 18]. Thus, sleep inertia severity may carry unique importance as a characteristic of focus in the screening and management of cognitive problems across many disorders and the broader population; however, to our knowledge, the relationships between sleep inertia and daytime cognitive performance have not been evaluated.

This exploratory investigation was designed to address this knowledge gap, by examining relationships between subjective sleep inertia severity and performance on six widely used, validated cognitive tests in a population-based sample. We hypothesized that greater sleep inertia severity would be associated with poorer cognitive performance, which, if supported, would serve as an impetus for lines of future research that clarify the role of sleep inertia in cognitive health and aging.

## Methods

### Dataset description and sample formation

This exploratory, cross-sectional investigation analyzed data from participants recruited through the Wisconsin Sleep Cohort (WSC) study. The WSC is an ongoing, longitudinal study of sleep health. Comprehensive information on its design, methodology, and purpose is detailed elsewhere [19, 20]. Although the WSC has been following participants since the late 1980s, the first assessment of sleep inertia, the Sleep Inertia Questionnaire (SIQ) was included in data collection beginning in 2019. Spanning data collection years 2019–2023, WSC participants completed the SIQ [21], as well as the Hypersomnia Severity Index (HSI) [22] and Epworth Sleepiness Scale (ESS) [23]. During this window, participants were also asked to complete a standardized neurocognitive battery (detailed below). For inclusion in this analysis, participants had to complete the SIQ, HSI, ESS, and neurocognitive battery. An initial analytic sample was formulated with data from 462 adults. One participant was removed from the final analytic sample (*N* = 461) due to self-reported absence of sleep on the night prior to testing, which prevented an accurate estimate of the number of hours between self-reported awakening time and neurocognitive testing start time.

All participants provided informed consent, and all study procedures were approved by the University of Wisconsin-Madison Health Sciences Institutional Review Board.

### Collected measures

#### Cognitive tasks

Participants completed six cognitive tasks as part of the standardized, neurocognitive battery that had previously been utilized in the WSC: the Rey Auditory Verbal Learning Test (AVLT) [24, 25], Grooved Pegboard (GPB) [26, 27], Trail Making Test Part B (TMT-B) [28, 29], Symbol Digit Modalities Test (SDMT) [30, 31], Controlled Oral Word Association Test (COWAT) [32, 33], and Digit Cancellation Test (D-CAT) [34]. Generally, participants completed the neurocognitive battery in 35 minutes. Initially, participants completed the AVLT, followed by the GPB, TMT-B, SDMT, COWAT, and D-CAT, with these subsequent tasks administered sequentially and in a standardized fashion targeting a 30-min completion timeframe. Following these tasks, participants completed the AVLT long-delayed recall. In situations when tasks could not be administered within the 30-min time period (e.g., due to logistical or participant-level issues), the remaining components of the battery were conducted following the long-delayed recall portion of the AVLT. The neurocognitive battery was collected during typical waking hours, not immediately upon awakening, and typically in the late morning/mid-afternoon or evening depending on participant availability and other study protocols.

A total of seven outcomes were analyzed from the six cognitive tasks, including both immediate and delayed recall from the AVLT. Full descriptions of the tasks and outcomes used in the investigation are available in Supplementary Table 1.

#### Hypersomnolence measures

Participants completed three validated, self-report somnolence measures: Sleep Inertia Questionnaire [21], Epworth Sleepiness Scale [23], and Hypersomnia Severity Index [22]. These questionnaires were administered prior to the neurocognitive assessment. Descriptions of the measures and their outcomes are detailed below.

##### Sleep Inertia Questionnaire (SIQ)

The SIQ is a 23-item self-report questionnaire that is used to assess cognitive, behavioral, emotional, and physiological correlates of sleep inertia [21]. The first 21 items are scored on a 5-point Likert scale (1–5). The total scores range from 21 to 105, with higher scores indicating greater sleep inertia severity. Items #22 and 23 are open response questions, inquiring about the duration of time for which symptoms persist in a given day (minutes), as well as the number of days per week that the symptoms present (days). In scoring the SIQ for these analyses, items #22 and 23 were not included. For the outcome of this study, the summation of the first 21 items was used to calculate the total SIQ score. Previous research using factor analysis has identified four subscales of the SIQ: Physiological, cognitive, emotional, and responses to sleep inertia (“responses”) [21]. Summed totals for each of the factors were computed for secondary analyses.

##### Epworth Sleepiness Scale (ESS)

The ESS is a widely utilized self-report questionnaire, serving as the gold standard measure of subjective daytime sleepiness [23]. The ESS consists of 8 items that are each scored on a 4-point Likert scale (0–3). The total scores range from 0 to 24, with higher scores indicating greater daytime sleepiness. For this study, the total score across the 8 items was used.

##### Hypersomnia Severity Index (HSI)

The HSI is a 9-item self-report questionnaire to measure the severity of hypersomnia symptoms and related impairment [22]. Each item is scored on a 5-point Likert scale (0–4). The total scores range from 0 to 36, with higher scores indicating greater severity of hypersomnia symptoms. For this study, the total score across the 9 items was used.

#### Additional covariates: demographics and characteristics

Beyond the cognitive tasks and hypersomnolence measures, demographic and other participant characteristics were used as covariates in the statistical models. These included age at time of neurocognitive battery, sex, highest education level, current alcohol and caffeine use, and smoking history. Body mass index (BMI; kg/m^2^) was calculated based on measured height and weight. Participants also completed a modified Zung Self-Rating Depression Scale (ZSDS) [35], which has historically been utilized in prior WSC waves (sans item 6 regarding enjoyment of sex). A subsequent core depressive factor [36] (ZSDS-D) was calculated using available items. A variable was also computed that captured the duration of time between self-reported awakening on testing day and the start time of neurocognitive testing (“hours since awakening on testing day”; hours).

Age (years), BMI, hours since awakening on testing day, and the ZSDS-D were modeled as continuous variables, while sex (female vs. male), education level (high school or less/some college or more), alcohol use (< 1, 1–5, and ≥ 5 drinks per week), caffeine use (< 1, 1–3, and ≥ 3 cups per day), and smoking history (never, past, and current) were included in analyses as categorical variables.

Circadian preference was determined based on responses to the self-morningness/eveningness (Self-ME) question [37], which is the last question from the Horne and Östberg morningness-eveningness questionnaire [38]. The single-item question asks whether the participant considered themselves to be a morning or evening person, with four response options: Definitely a morning type, more of a morning type than evening type, more of an evening type than a morning type, definitely an evening type. Circadian preference was modeled as a categorical variable including all four response levels.

Participants completed overnight, nocturnal polysomnography (PSG) as part of their participation in the WSC, with some undergoing multiple PSG studies given the longitudinal nature of the study. Apnea–hypopnea index (AHI; average number of apneas and hypopneas per hour of sleep) was determined from the PSG closest in time to neurocognitive assessment, and categorized (< 5, 5– < 15, and ≥ 15 events/hour as absence of, mild, or moderate or worse obstructive sleep apnea). Participants who utilized CPAP at the time of PSG were included in the moderate or worse OSA category. The time between the closest PSG and the neurocognitive assessment varied by participant; therefore, a covariate was used to adjust for the time between these study measures (years). Habitual sleep duration was self-reported and also utilized as a categorical variable (≤ 6 h, 6–9 h, and ≥ 9 h).

### Statistical analyses

Descriptive statistics were computed for the primary analytic sample. Sample means ± standard deviation are reported for continuous variables, with proportions and counts provided across levels of categorical variables. In primary analyses, plot diagnostics were inspected, including the assessment of the distribution of residuals. Normal residual distributions were observed and linear regression was employed to examine relationships between total scores from the hypersomnolence measures (SIQ, ESS, and HSI) and outcomes from the six cognitive tasks. Unadjusted and adjusted analyses were performed, with adjusted models controlling for age, sex, BMI, educational level, ZSDS-D, alcohol use per week, caffeine use per day, smoking history, circadian preference, habitual sleep duration, AHI, duration between PSG collection visit and neurocognitive assessment (years), and hours since awakening on testing day. Supplementary analyses assessed relationships between SIQ subscales and cognitive outcomes, with the adjusted models accounting for the covariates listed. For each regression, the regression coefficient (β), standard error (SE), and corresponding *p* value are reported. Data were analyzed using SAS software (SAS Institute Inc., Cary, NC), with *α* = 0.05 (two-sided) for statistical significance.

## Results

### Sample characteristics

Table 1 presents the characteristics for the sample, which included a total of 461 participants (average age = 73.84 ± 6.66 years), with a slightly higher proportion of males (55.8%) than females. The sample was predominantly non-Hispanic white (95.2%), with the majority of participants having some college education (76.6%). Average BMI of the sample fell within the obese category (31.1 ± 6.94), similar to U.S. population trends [39]. Caffeine and alcohol consumption varied across the sample. The majority of participants were never smokers (55.8%), and there were few active smokers in the sample, though 40.6% of the sample endorsed a past history of smoking.

**Table 1:** Sample characteristics—primary analytic sample

Characteristic	Value
Sample size (*n*)	461
Age (years)	73.8 ± 6.66
Sex	
* Female*	204 (44.3%)
* Male*	257 (55.8%)
Race/ethnicity	
* White*	439 (95.2%)
*American Indian or Alaska Native*	8 (1.74%)
*Hispanic or Latino*	5 (1.08%)
*Black or African American*	5 (1.08%)
*Asian*	4 (0.87%)
Body mass index (BMI; kg/m^2^)	31.1 ± 6.94
Education history	
*High school or less*	108 (23.4%)
*Some college or more*	353 (76.6%)
Caffeine use per day	
< *1 serving*	72 (15.6%)
≥ *1 and* < *3 servings*	221 (47.9%)
≥ *3 servings*	168 (36.4%)
Alcohol use per week	
< *1 drink*	188 (40.8%)
≥ *1 and* < *5 drinks*	144 (31.2%)
≥ *5 drinks*	129 (28.0%)
Smoking history	
*Never*	257 (55.8%)
*Past*	187 (40.6%)
*Current*	17 (3.70%)
Apnea–hypopnea index (AHI; events per hour)	
*AHI* < *5*	171 (37.1%)
*5* ≤ *AHI* < *15*	130 (28.2%)
*AHI* ≥ *15 or CPAP use during polysomnography*	160 (34.7%)
Self-report habitual sleep duration (hours)	
≤ *6*	70 (15.2%)
> *6 and* < *9*	341 (74.0%)
≥ *9*	50 (10.9%)
Zung Self-Rating Depression Scale, core depressive factor	10.6 ± 3.39
Hours between self-reported waketime neurocognitive battery start time	10.9 ± 2.50
Chronotype	
*Definitely morning*	134 (29.1%)
*More morning than evening*	190 (41.2%)
*More evening than morning*	93 (20.2%)
*Definitely evening*	44 (9.5%)

Characteristics for the primary analytic sample (*n* = 461)

In terms of self-reported circadian preference, the majority reported morning preference (70.3%) compared to evening preference (29.7%). For habitual sleep duration, most participants reported sleeping more than six and less than 9 h per night (74.0%), with 15.2% and 10.9% reporting sleeping ≤ 6 h and ≥ 9 h, respectively. Based on our criteria, 37.1% of the sample did not have significant sleep-disordered breathing, while 28.2% had mild and 34.7% had moderate-to-severe sleep apnea. Additionally, 42 individuals wore CPAP during the PSG used in these analyses.

Regarding the timing of the neurocognitive battery, the earliest and latest start times were 11:52 and 19:30, respectively. 20.3% of the sample was administered the neurocognitive battery prior to 17:00, with the neurocognitive battery administered after 17:00 for 70.7% of the sample. On average, neurocognitive testing started 10.9 ± 2.50 h after self-reported waketime on testing day. The mean time difference between polysomnography (to quantify presence or absence of OSA) and the neurocognitive battery was 10 years ± 4, with a range of 4 to 31 years.

Means and standard deviations of hypersomnolence measures and cognitive tasks are provided in Supplementary Table 2.

### Associations between hypersomnolence measures and cognitive tests

Table 2 presents the unadjusted and adjusted analyses between total scores of the hypersomnolence measures and cognitive tests.

**Table 2:** Relationships between hypersomnolence measures and cognition

Cognitive test		Unadjusted		Adjusted	
		*β (SE)*	*p*	*β (SE)*	*p*
Sleep inertia score					
	AVLT–immediate	− 0.03 (0.05)	0.51	− 0.02 (0.05)	0.78
	AVLT–delayed	− 0.02 (0.02)	0.29	− 0.02 (0.02)	0.23
	GPB	**1.48 (0.41)**	**0.0003**	**1.30 (0.43)**	**0.003**
	TMT-B	**0.95 (0.27)**	**0.0005**	**0.93 (0.29)**	**0.001**
	SDMT	** − 0.12 (0.05)**	**0.02**	− 0.09 (0.05)	0.07
	COWAT	− 0.10 (0.06)	0.11	− 0.06 (0.07)	0.43
	D-CAT	1.17 (0.63)	0.06	0.28 (0.68)	0.68
Epworth sleepiness scale					
	AVLT–immediate	0.01 (0.13)	0.94	0.17 (0.13)	0.17
	AVLT–delayed	− 0.02 (0.05)	0.74	0.02 (0.05)	0.59
	GPB	1.52 (1.09)	0.16	0.18 (1.02)	0.86
	TMT-B	1.29 (0.72)	0.07	0.86 (0.67)	0.20
	SDMT	− 0.15 (0.13)	0.25	− 0.06 (0.12)	0.60
	COWAT	− 0.30 (0.16)	0.07	− 0.26 (0.16)	0.11
	D-CAT	3.07 (1.66)	0.07	2.15 (1.58)	0.17
Hypersomnia severity index					
	AVLT–immediate	0.13 (0.09)	0.18	**0.21 (0.10)**	**0.03**
	AVLT–delayed	0.03 (0.03)	0.39	0.04 (0.04)	0.26
	GPB	1.47 (0.78)	0.06	0.68 (0.79)	0.39
	TMT-B	0.33 (0.52)	0.53	0.05 (0.53)	0.92
	SDMT	− 0.11 (0.10)	0.27	− 0.01 (0.09)	0.88
	COWAT	−0.03 (0.12)	0.79	0.05 (0.13)	0.67
	D-CAT	0.73 (1.20)	0.54	− 0.70 (1.24)	0.57

The unadjusted and adjusted analyses between hypersomnolence measures and cognitive tests. Hypersomnolence measures include the total scores from Sleep Inertia Questionnaire, Epworth Sleepiness Scale, and Hypersomnia Severity Index, with scores ranging from 21 to 105, 0 to 24, and 0 to 36, respectfully. Cognitive outcomes include results from the; *(AVLT; Immediate and Delayed)* auditory verbal learning test, *GPB* grooved pegboard, *TMT-B* trail making test – part B, *SDMT* symbol digit modalities test, *COWAT* controlled oral word association test, and *D-CAT* digit cancellation test. Adjusted models accounted for BMI, Zung Self-Rating Depression Scale-Core Depressive Factor, sex, education, alcohol use per week, caffeine use per day, smoking history, habitual sleep duration, chronotype, polysomnography AHI, and cognitive testing time of day. Regression coefficient (*β*), standard error (*SE*), and associated *p* value are included from each analysis

Statistically significant findings (*p*<0.05) are in bold

In unadjusted analyses, greater sleep inertia severity was significantly associated with slower completion time on the GPB (*β* = 1.48, *SE* = 0.41, *p* = 0.0003) and TMT-B (*β* = 0.95, *SE* = 0.27, *p* = 0.0005), as well as less correct matched numbers on the SDMT (*β* = − 0.12, *SE* = 0.05, *p* = 0.02). Significant associations were also observed in adjusted analyses for the relationship between SIQ and GPB (*p* = 0.003) and SIQ and TMT-B (*p* = 0.001), while trend-level significance was observed between SIQ and SDMT (*p* = 0.07).

For ESS and HSI, no significant associations were observed with cognitive outcomes in unadjusted analyses. A statistically significant association was observed only in adjusted analyses between HSI and AVLT–immediate recall (*β* = 0.21, *SE* = 0.10, *p* = 0.03), suggesting that greater hypersomnia symptom severity was associated with more correctly recalled words for the immediate recall trial.

### Associations between SIQ subscales and cognitive tests

Table 3 presents the unadjusted and adjusted analyses between the SIQ Subscales (physiological, cognition, response, emotion) and the neurocognitive battery.

**Table 3:** Relationships between SIQ subscales and cognition

Cognitive test		Unadjusted		Adjusted	
		*β (SE)*	*p*	*β (SE)*	*p*
Physiological					
	AVLT–immediate	− 0.13 (0.10)	0.20	− 0.02 (0.11)	0.84
	AVLT–delayed	− 0.05 (0.04)	0.17	− 0.03 (0.04)	0.37
	GPB	**4.61 (0.81)**	** < 0.0001**	**3.88 (0.84)**	** < 0.0001**
	TMT-B	**2.42 (0.54)**	** < 0.0001**	**1.94 (0.57)**	**0.0008**
	SDMT	** − 0.37 (0.10)**	**0.004**	** − 0.25 (0.10)**	**0.01**
	COWAT	** − 0.27 (0.13)**	**0.03**	− 0.11 (0.14)	0.44
	D-CAT	**3.76 (1.28)**	**0.004**	1.52 (1.35)	0.26
Cognition					
	AVLT–immediate	** − 0.30 (0.15)**	** < 0.05**	− 0.13 (0.16)	0.39
	AVLT–delayed	** − 0.13 (0.05)**	**0.01**	− 0.10 (0.06)	0.07
	GPB	**5.21 (1.24)**	** < 0.0001**	**3.51 (1.25)**	**0.005**
	TMT-B	**2.95 (0.82)**	**0.0004**	**1.93 (0.83)**	**0.02**
	SDMT	** − 0.51 (0.15)**	**0.0008**	− 0.26 (0.15)	0.08
	COWAT	− 0.16 (0.19)	0.41	0.05 (0.20)	0.80
	D-CAT	**4.52 (1.91)**	**0.02**	1.20 (1.96)	0.54
Response					
	AVLT–immediate	0.29 (0.14)	0.04	0.09 (0.15)	0.57
	AVLT–delayed	0.07 (0.05)	0.16	− 0.02 (0.05)	0.78
	GPB	− 1.89 (1.22)	0.12	− 0.90 (1.23)	0.46
	TMT-B	− 0.24 (0.80)	0.76	0.93 (0.80)	0.25
	SDMT	0.27 (0.15)	0.07	0.07 (0.14)	0.64
	COWAT	− 0.13 (0.18)	0.47	− 0.21 (0.20)	0.29
	D-CAT	− 1.74 (1.86)	0.35	− 1.54 (1.90)	0.42
Emotion					
	AVLT–immediate	− 0.46 (0.37)	0.22	− 0.45 (0.41)	0.27
	AVLT–delayed	− 0.07 (0.13)	0.59	− 0.07 (0.15)	0.63
	GPB	**7.58 (3.09)**	**0.01**	**6.82 (3.25)**	**0.04**
	TMT-B	**5.63 (2.03)**	**0.01**	**5.78 (2.15)**	**0.007**
	SDMT	− 0.56 (0.38)	0.14	− 0.32 (0.38)	0.40
	COWAT	− 0.70 (0.47)	0.13	− 0.86 (0.52)	0.10
	D-CAT	3.54 (4.75)	0.46	0.35 (5.07)	0.94

The unadjusted and adjusted analyses between subscales of the Sleep Inertia Questionnaire and cognitive tests. The subscales of the Sleep Inertia Questionnaire include physiological, cognitive, response, and emotion. Cognitive outcomes include results from the; *(AVLT; Immediate and Delayed)* auditory verbal learning test, *GPB* grooved pegboard, *TMT-B* trail making test – part B, *SDMT* symbol digit modalities test, *COWAT* controlled oral word association test, and *D-CAT* digit cancellation test. Adjusted models accounted for BMI, Zung Self-Rating Depression Scale–Core Depressive Factor, sex, education, alcohol use per week, caffeine use per day, smoking history, habitual sleep duration, chronotype, polysomnography AHI, and cognitive testing time of day. *β* Regression coefficient, *SE* standard error, and associated *p* value are included from each analysis

Statistically significant findings (*p*<0.05) are in bold

For the physiological subscale, higher severity was significantly associated with slower completion time on the GPB (*β* = 4.61, *SE* = 0.81, *p* < 0.0001), TMT-B (*β* = 2.42, *SE* = 0.54, *p* < 0.0001), and D-CAT (*β* = 3.76, *SE* = 1.28, *p* = 0.004), as well as fewer correctly matched symbols on the SDMT (*β* = −0.37, *SE* = 0.10, *p* = 0.004) and total words on the COWAT (*β* = −0.27, *SE* = 0.13, *p* = 0.03). Statistically significant associations were also observed in adjusted analyses for the GPB (*p* < 0.0001), TMT-B *(p* = 0.0008), and SDMT (*p* = 0.01).

For the cognition subscale, higher severity significantly associated with slower completion time on the GPB (*β* = 5.21, *SE* = 1.24, *p* < 0.0001), TMT-B (*β* = 2.95, *SE* = 0.82, *p* = 0.0004), and D-CAT (*β* = 4.52, *SE* = 1.91, *p* = 0.02), as well as fewer correctly matched symbols on the SDMT (*β* = −0.51, *SE* = 0.15, *p* = 0.0008) and total words on the AVLT – Immediate (*β* = −0.30, *SE* = 0.15, *p* < 0.05). Statistically significant associations were also observed in adjusted analyses for the GPB (*p* = 0.005) and TMT-B *(p* = 0.02).

For the emotion subscale, higher severity was significantly associated with slower completion time on the GPB (*β* = 7.58, *SE* = 3.09, *p* = 0.01) and TMT-B (*β* = 5.64, *SE* = 2.03, *p* = 0.01), with statistically significant associations also observed in adjusted analyses for both GPB (*p* = 0.04) and TMT-B *(p* = 0.007).

For the response subscale, no statistically significant relationships were observed in unadjusted or adjusted analyses.

### APOE4 sensitivity analysis

APOE4 positive status was not available for all participants; thus, APOE4 was not included as a covariate in primary analyses. However, given established relationships between APOE4 and cognitive functioning [40, 41], a sensitivity analysis was performed using similar methodology that examined relationships between SIQ (total and subscales) and cognitive tasks using 389 WSC participants from the primary sample for whom APOE4 status was available. Adjusted analyses were performed, including all covariates accounted for in the primary analyses, as well as a dichotomized categorical variable reflecting APOE4 status (no alleles vs. any alleles).

The results from this analysis are presented in Supplementary Table 3. Overall, the associations between SIQ and cognitive outcomes did not meaningfully change in these analyses, relative to the primary analyses.

### Nightshift worker sensitivity analysis

To assess whether inclusion of night shift workers influenced the primary findings, a sensitivity analysis was also conducted excluding participants who reported actively being a night shift worker at time of assessment (*n* = 13), yielding a final analytic sample of 448 individuals. Results of this sensitivity analysis are presented in Supplementary Table 4. Overall, the associations between SIQ and cognitive measures did not substantively change relative to primary analyses.

## Discussion

This exploratory investigation was designed to advance the understanding of the relationship between sleep inertia severity, a common, more specific symptom that often (but not always) occurs with other hypersomnolence symptoms, and cognition, by examining associations between the Sleep Inertia Questionnaire (SIQ) and six widely utilized measures of cognition. Greater sleep inertia severity was significantly associated with worse cognition, particularly on the Grooved Pegboard (GPB) and Trail Making Test–Part B (TMT-B) tasks. The results also suggest that sleep inertia, as measured by the SIQ, may be a uniquely important characteristic of hypersomnolence in the context of cognitive performance, especially given that neither the Epworth Sleepiness Scale (ESS) nor the Hypersomnia Severity Index (HSI) consistently correlated with cognitive outcomes. These results highlight the potential importance of sleep inertia as a specific, person-level sleep factor that may be more strongly associated with cognitive performance in older adults compared to other sleep-related symptoms.

The null findings related to the ESS are somewhat unexpected, given prior evidence linking daytime sleepiness to impaired cognition and increased risk for cognitive problems [42, 43]. However, prior research has not consistently demonstrated significant relationships between the ESS and cognitive outcomes [44], which may reflect both limitations of the ESS as the gold-standard, self-report measure of daytime sleepiness [45, 46] and methodological variations across studies. For the HSI, a single, weak (*p* = 0.03) significant association was observed in the adjusted models, which associated hypersomnia symptom severity with performance on the Rey Auditory Verbal Learning Test (AVLT). The direction of this association (*β* = 0.21) indicated that greater hypersomnia symptom severity was associated with better verbal learning performance, which is seemingly paradoxical and difficult to explain. Overall, the totality of evidence suggests that sleep inertia, as measured by the SIQ, is more tightly connected to cognitive performance than the other measures of daytime sleepiness and related impairment considered in this investigation.

Across the investigation, sleep inertia severity consistently showed the strongest associations with the GPB and TMT-B tests, suggesting a link between sleep inertia and specific cognitive domains, particularly psychomotor speed and executive functioning. These tests also demonstrated consistent, significant associations with the SIQ’s physiological, cognitive, and emotional subscales, but not with the response subscale. This latter finding, while unexpected, may reflect the fact that many participants were retired and less likely to engage in behaviors measured by the response subscale (e.g., waking to an alarm). Notably, the overlap in cognitive functions assessed by GPB and TMT-B further supports a strong connection between sleep inertia and these specific cognitive processes.

Currently, the mechanisms underlying sleep inertia are not fully understood and are considered multifactorial, with neurophysiological, circadian, and cognitive factors involved [13]. It has been broadly hypothesized that the habituality and severity of pathological sleep inertia may be reflective of underlying dysregulation of the neural mechanisms that govern sleep–wake transitions [10]. Converging evidence supports the view that excessive sleep inertia represents a delayed or blunted transition from sleep to wake, which itself is a complex and understudied neurophysiological process. Proposed explanations include the slowed dissipation of sleep-promoting substances such as adenosine following awakening [10, 47], as well as disruptions in cortical arousal and prefrontal functioning that transiently impairs executive functioning [10, 13]. Additionally, more recent work has further implicated circadian misalignment and homeostatic processes, with sleep inertia severity exacerbated by awakening during the biological night or from slow-wave sleep [11, 48, 49]. Thus, sleep inertia is likely a multifactorial process that may be conceptualized as a dynamic interaction between neurochemical clearance, regional brain-state transitions, homeostatic factors, and circadian timing.

While the biological underpinnings that connect our observed associations between sleep inertia and cognition cannot be determined by this study, there are several lines of evidence that suggest sleep inertia may be plausibly associated with risk of neurodegenerative conditions. Broadly, clinical symptoms of disordered vigilance state regulation are frequently observed in both synucleinopathies and tauopathies, likely reflecting neuronal damage to circuits that normally segregate brain states [50, 51]. Emerging evidence suggests that cortical activity upon awakening from sleep follows a consistent spatio-temporal gradient hypothesized to reflect discharge patterns of arousal-related structures, including the locus coeruleus [52]. While transitions from sleep to wake are typically characterized by rapid activation of brain stem arousal systems with delayed activation of cortical regions and loss of brain functional segregation between task-positive (dorsal attention, salience, sensorimotor) and task-negative (default mode) networks that result in sleep inertia [53, 54], more prolonged episodes could in theory be in part mediated by impaired monoaminergic neurotransmission upon or after awakening [55, 56]. Notably, the locus coeruleus, the primary noradrenergic nucleus in the central nervous system, is a key regulator of both transitions from sleep to wake, as well as between REM and NREM sleep [57, 58]. In this context, it is notable that the locus coeruleus is one of the earliest sites of pathological tau accumulation, becoming hypoactive in the pre-clinical stages of Alzheimer’s disease (AD) [59]. Interestingly, caffeine, an adenosine receptor antagonist [60], has been demonstrated to reduce sleep inertia in sleep deprivation paradigms as well as increase activity in the locus coeruleus after administration [47, 61]. While speculative, emerging data that link caffeine use, an established sleep inertia countermeasure, to diminished AD risk and improved cognitive function [62] may indirectly connect the findings of this investigation to other epidemiological studies of AD risk. Future research that examines connections between these biological factors, objective measures of sleep inertia, and risk of neurodegenerative conditions is thus likely to be a fruitful area of inquiry.

Despite several strengths of this investigation, which include a large, well-characterized population-based dataset that also includes objective measures (i.e., obstructive sleep apnea severity and APOE4 status utilized in sensitivity analyses), this investigation has limitations that merit consideration. Although the WSC provides rich and robust data, the sample characteristics limit the generalizability of the findings. The sample was predominantly white, non-Hispanic older adults. Moreover, as a cross-sectional study, longitudinal research, preferably with more diverse samples, is needed to explore the links between sleep inertia and cognition across the lifespan. The majority of cognitive assessments were performed in the evenings, which may have influenced neurocognitive outcomes. However, statistical models accounted for hours since awakening on testing day which reduces this as a potential confounder, and further underscores the importance of the observation that measured performance on neurocognitive testing was unlikely to be the direct result of severe sleep inertia occurring while testing was performed. Moreover, although models adjusted for hours since awakening on the day of testing and chronotype, this study was not designed to evaluate whether these factors moderate the associations between sleep inertia (and other focal predictors) and neurocognitive outcomes. Future research is needed to more fully characterize potential interactive effects between sleep inertia, time since awakening, chronotype, and cognitive performance. Additionally, although the SIQ is a validated measure of sleep inertia severity, it relies on retrospective self-reporting of features related to sleep inertia. Leveraging ambulatory, objective assessments could enhance measurement precision and validity, and will be an important next step to advance this emerging area of inquiry. Lastly, given the exploratory nature of these analyses, we did not correct for multiple comparisons. Therefore, the number of comparisons conducted increases the possibility that some findings may reflect Type I error rather than true effects. Future studies are thus clearly needed to replicate and advance this line of inquiry.

The results of this investigation connect the symptom of sleep inertia with cognitive performance in testing performed not immediately upon awakening. While these results should be considered preliminary and require replication in other samples, they do raise the intriguing possibility that sleep inertia may be a key risk factor for cognitive decline across aging. As such, sleep inertia complaints among older adults could prove to be a harbinger of cognitive decline that could be readily incorporated into clinical care assessments and care algorithms. Additionally, evaluation of treatments designed to reduce sleep inertia and their downstream effects on daytime cognitive performance could prove to be an important area of future investigation to modify cognitive trajectories in older adults.

## Supplementary information

Below is the link to the electronic supplementary material.

ESM 1 (DOCX 31.5 KB)

## Data Availability

The data supporting these findings is available from the corresponding author upon reasonable request, and in concordance with required IRB approval.
