# Correction: Association between sleep inertia and cognitive performance among older adults in the Wisconsin Sleep Cohort study

**DOI:** 10.1007/s44470-026-00148-x

**Published:** 2026-07-20

**Authors:** Jessica J. Love, Jesse D. Cook, Erika W. Hagen, Amanda Rasmunon, Laurel A. Ravelo, Mari Palta, Paul E. Peppard, David T. Plante

**Affiliations:** 1 Department of Psychiatry, School of Medicine and Public Health, University of Wisconsin–Madison, Madison, WI USA; 2 Department of Population Health Sciences, School of Medicine and Public Health, University of Wisconsin–Madison, Madison, WI USA; 3 Department of Psychology, University of Wisconsin– Madison, Madison, WI USA

**Correction: Journal of Clinical Sleep Medicine (2026) 22:105**

10.1007/s44470-026-00133-4

The original online version of this article was revised to denote that bold data in Tables 2 and 3 indicated statistically significant findings (p<0.05).

The original article has been corrected.

