# Supplementary material for: Association between sleep inertia and cognitive performance among older adults in the Wisconsin Sleep Cohort study
Source: J Clin Sleep Med. 2026 Jul 6;22(1):105. doi: 10.1007/s44470-026-00133-4 (PMC13337965; doi:10.1007/s44470-026-00133-4)
Supplement: Supplementary file 1 — (DOCX 31.5 KB) [file 44470_2026_133_MOESM1_ESM.docx]

**Supplementary Information (SI)**

**Article Title:** Association between Sleep Inertia and Cognitive Performance Among Older Adults in the Wisconsin Sleep Cohort Study

**Journal Name:** Journal of Clinical Sleep Medicine

**Author Names:** Jessica J. Love, BA, Jesse D. Cook, PhD, Erika W. Hagen, PhD, Amanda Rasmunon, MS, Laurel A. Ravelo, MA, Mari Palta, PhD, Paul E. Peppard, PhD, David T. Plante, MD, PhD

**Corresponding Author Information:**

David T. Plante, MD, PhD

University of Wisconsin – Madison, School of Medicine and Public Health, Department of Psychiatry, Madison, WI, USA

University of Wisconsin – Madison, Department of Psychology, Madison, WI, USA

[dplante@wisc.edu](mailto:dplante@wisc.edu)

**Supplementary Table S1.** Cognitive Tests in the Neurocognitive Battery – Description and Investigation Outcomes

| **Cognitive Test** | **Description and Outcomes** |
| --- | --- |
|  |  |
| Rey Auditory Verbal Learning Test (AVLT): Immediate and Delayed | The AVLT is a verbal learning and memory task. Individuals are read a list of 15 semantically unrelated words (List A), with individuals immediately prompted to recall words that they can remember [31, 32]. This process is completed five times consecutively, with the score from each trial reflecting the total number of correctly recalled words. For the sixth trial, a new list of 15 words (List B) is read to the participants, with participants asked to recall words from List A. After completion of the sixth trial, participants undergo at-least a 15-minute delay before a long-delayed recall of List A, with 30-minutes afforded to participants in this study. Both the immediate and delayed recall from the AVLT were included as outcomes. Immediate recall reflected the total sum of correct responses from the first five trials for List A, delayed recall reflected performance for the single trial after the 30-minute delay. |
|  |  |
|  |  |
| Grooved Pegboard  (GPB) | The GPB is a commonly utilized task to assess motor control and dexterity, psychomotor speed, and visuomotor coordination [33, 34]. Briefly, the task involves a 5 x 5 apparatus that has 25 holes with unique grooves. Participants are tasked with inserting pegs sequentially into the holes, fitting the pegs by rotating the grooves so that they align with the hole. Participants complete the task both right- and left-handed, with time to completion compiled from each trial. Participants complete the task from top to bottom, moving left to right when using the right hand (right to left when using the left hand). Participants initially complete the test using their dominant hand, followed by their nondominant hand. The total sum time across both trials served as the outcome. |
|  |  |
|  |  |
| Trail Making Test –  Part B (TMT-B) | The TMT-B is one of the most commonly used cognitive assessments, with performance used to measure cognitive flexibility and speed, as well as serving as a proxy for global executive functioning [35, 36]. Briefly, participants are tasked with drawing lines to connect numbered and lettered circles, alternating alphanumeric sequencing (i.e., 1-A-2-B-3-C) until reaching the letter L. If a participant makes a mistake during the test, then the administrator directs the participant to the most recent correct answer. The total completion time (seconds) was used as the outcome. |
|  |  |
|  |  |
| Symbol Digit Modalities Test (SDMT) | The SDMT is a commonly utilized cognitive measure in research and clinical practice. Research has indicated that the SDMT is a measure of multiple cognitive processes, including cognitive processing speed, memory, and lexical access speed [37, 38]. Briefly, participants are provided a reference key with nine numbers (1 through 9) corresponding to nine symbols. Participants are given the opportunity to practice matching the correct number with the appropriate symbol before being tasked with matching as many numbers to symbols as possible within 90 seconds. The total correct number of matched numbers was used as the outcome. |
|  |  |
|  |  |
| Controlled Oral Word Association Test  (COWAT) | The COWAT is a widely used test to assess verbal fluency and executive functioning, often leveraged as a measure in the context of cognitive impairment and neurocognitive disorders [39, 40]. Briefly, participants are asked to produce as many words as possible that begin with a given letter (i.e., F) within a 1-minute time period, with instruction to exclude proper nouns, numbers, and the same word with a different suffix. This is repeated three times, with a different letter used each time. In the collection of this dataset, the letters C, F, and L were used across trials, respectively. The total number of viable words provided across the three trials was used as the outcome. |
|  |  |
|  |  |
| Digit Cancellation Test  (D-CAT) | The D-CAT is a task that is commonly utilized to assess the attention, processing speed, and executive functioning [41]. Participants are provided a sheet of paper filled with a matrix of numbers. The participant is asked to cross out a specific number (i.e., 6) when it appears in the matrix, working left-to-right across the sheet and moving through rows sequentially. This process is repeated twice, first with the numbers in blue ink and the second with the numbers in red ink. The summed total time (seconds) across both trials was used as the outcome. |
|  |  |

**Supplementary Table S2.** Means and Standard Deviations Across Hypersomnolence Measures and Cognitive Tests

| **Measure and Cognitive Test** | | **Mean ± Standard Deviation** |
| --- | --- | --- |
|  | |  |
| **Hypersomnolence Measure** | |  |
|  | |  |
| Epworth sleepiness scale | | 6.85 ± 3.57 |
| Hypersomnia severity index | | 7.21 ± 4.96 |
| Sleep inertia questionnaire: | |  |
|  | Total | 31.7 ± 9.38 |
|  | Physiological | 12.6 ± 4.63 |
|  | Cognition | 7.49 ± 3.11 |
|  | Response | 8.32 ± 3.21 |
|  | Emotion | 3.67 ± 1.25 |
|  | |  |
|  | |  |
| **Cognitive Test** | |  |
|  | |  |
| AVLT – Immediate (N) | | 42.8 ± 10.0 |
| AVLT – Delayed (N) | | 8.24 ± 3.55 |
| GPB (seconds) | | 204 ± 83.3 |
| TMT-B (seconds) | | 99.8 ± 54.9 |
| SDMT (N) | | 43.9 ± 10.2 |
| COWAT (N) | | 42.3 ± 12.5 |
| D-CAT (seconds) | | 447 ± 127 |
|  | |  |

**Supplementary Table 2.** Means and standard deviations across hypersomnolence measures and cognitive tests from the primary analytic sample. Hypersomnolence measures include the sleep inertia questionnaire (SIQ), Epworth sleepiness scale (ESS), and hypersomnia severity index (HSI). Total scores are reported for the SIQ, ESS, and HSI, with scores additionally provided for the SIQ subscales: Physiological, Cognition, Response, and Emotion. Cognitive tests include the Rey Auditory Verbal Learning Test (AVLT; Immediate and Delayed), Grooved Pegboard Task (GPB), Trail Making Test – Part B (TMT-B), Symbol Digit Modalities Test (SDMT), Controlled Oral Word Association Test (COWAT), and Digit Cancellation Test (D-CAT).

**Supplementary Table S3.** Sensitivity Analyses: Adjusted models accounting for APOE4 status

| **Measure** | | ***β* (*SE*)** | | | | | | |
| --- | --- | --- | --- | --- | --- | --- | --- | --- |
|  |  | **AVLT-I** | **AVLT-D** | **GPB** | **TMT-B** | **SDMT** | **COWAT** | **D-CAT** |
|  | |  |  |  |  |  |  |  |
| **SIQ** | |  |  |  |  |  |  |  |
|  | *Total Score* | -0.03 (0.06) | -0.03 (0.02) | **1.13 (0.46)*** | **1.06 (0.32)**** | **-0.12 (0.06)*** | -0.12 (0.08) | 0.56 (0.73) |
|  | *Physiological* | -0.04 (0.12) | -0.06 (0.04) | **3.40 (0.91)***** | **2.11 (0.64)**** | **-0.29 (0.12)*** | -0.22 (0.16) | 2.01 (1.47) |
|  | *Cognition* | -0.13 (0.18) | -0.11 (0.06) | **2.51 (1.34)*** | **2.25 (0.94)*** | -0.28 (0.17) | -0.003 (0.23) | 1.83 (2.14) |
|  | *Response* | -0.09 (0.15) | -0.02 (0.05) | -0.90 (1.23) | 0.93 (0.80) | 0.07 (0.14) | -0.21 (0.20) | -1.54 (1.90) |
|  | *Emotion* | -0.45 (0.41) | -0.07 (0.15) | **6.82 (3.25)*** | **5.78 (2.15)**** | -0.32 (0.38) | **0.86 (0.52)*** | 0.35 (5.07) |
|  | |  |  |  |  |  |  |  |
| **ESS** | | 0.18 (0.14) | 0.01 (0.05) | 0.15 (1.04) | 0.63 (0.73) | -0.09 (0.13) | -0.23 (0.17) | 2.31 (1.65) |
| **HSI** | | 0.19 (0.11) | 0.02 (0.04) | 0.91 (0.83) | 0.24 (0.58) | -0.11 (0.10) | -0.02 (0.14) | 0.13 (1.32) |
|  | |  |  |  |  |  |  |  |

**Supplementary Table 3.** Sensitivity analyses presenting results from comparisons between hypersomnolence measures and cognitive tests in a subsample of participants who had genetic information, affording the ability to additionally control for APOE4 status. Hypersomnolence measures include the sleep inertia questionnaire (SIQ), Epworth sleepiness scale (ESS), and hypersomnia severity index (HSI). Cognitive tests include the Rey Auditory Verbal Learning Test (AVLT; Immediate [I] and Delayed [D]), Grooved Pegboard Task (GPB), Trail Making Test – Part B (TMT-B), Symbol Digit Modalities Test (SDMT), Controlled Oral Word Association Test (COWAT), and Digit Cancellation Test (D-CAT). Values reflect the regression coefficient (β) and standard error (SE) from the adjusted regression that controlled for the same covariates as the primary analyses, along with APOE4 status. Statistical significance is denoted by * *p* < 0.05, ** *p* < 0.01, *** *p* < 0.001, with statistically significant associations (*p* < 0.05) bolded.

**Supplementary Table S4.** Sensitivity Analyses: Adjusted models accounting for Nightshift Workers

| **Measure** | | ***β* (*SE*)** | | | | | | |
| --- | --- | --- | --- | --- | --- | --- | --- | --- |
|  |  | **AVLT-I** | **AVLT-D** | **GPB** | **TMT-B** | **SDMT** | **COWAT** | **D-CAT** |
|  | |  |  |  |  |  |  |  |
| **SIQ** | |  |  |  |  |  |  |  |
|  | *Total Score* | -0.02 (0.06) | -0.02 (0.02) | **1.24 (0.44)**** | **0.91 (0.29)**** | -0.10 (0.05) | -0.08 (0.07) | 0.34 (0.70) |
|  | *Physiological* | -0.03 (0.11) | -0.04 (0.04) | **3.89 (0.86)***** | **1.98 (0.59)**** | **-0.27 (0.10)**** | -0.17 (0.14) | 1.66 (1.39) |
|  | *Cognition* | -0.17 (0.16) | -0.11 (0.06) | **3.78 (1.26)*** | **1.91 (0.85)*** | -0.28 (0.15) | -0.007 (0.20) | 1.40 (2.00) |
|  | *Response* | 0.12 (0.15) | -0.005 (0.06) | -1.55 (1.25) | 0.76 (0.83) | 0.08 (0.15) | -0.23 (0.20) | -1.54 (1.96) |
|  | *Emotion* | -0.35 (0.41) | -0.07 (0.15) | 6.05 (3.31) | **5.90 (2.20)*** | -0.35 (0.39) | **-0.80 (0.53)*** | 0.51 (5.21) |
|  | |  |  |  |  |  |  |  |
| **ESS** | | 0.17 (0.13) | 0.02 (0.05) | 0.10 (1.02) | 0.91 (0.68) | -0.06 (0.12) | -0.30 (0.16) | 2.03 (1.60) |
| **HSI** | | **0.21 (0.10)*** | 0.04 (0.04) | 0.50 (0.82) | -0.005 (0.55) | -0.02 (0.10) | 0.01 (0.13) | -0.57 (1.28) |
|  | |  |  |  |  |  |  |  |

**Supplementary Table 4.** Sensitivity analyses presenting results from comparisons between hypersomnolence measures and cognitive tests in a subsample of participants excluding nightshift workers at time of study. Hypersomnolence measures include the sleep inertia questionnaire (SIQ), Epworth sleepiness scale (ESS), and hypersomnia severity index (HSI). Cognitive tests include the Rey Auditory Verbal Learning Test (AVLT; Immediate [I] and Delayed [D]), Grooved Pegboard Task (GPB), Trail Making Test – Part B (TMT-B), Symbol Digit Modalities Test (SDMT), Controlled Oral Word Association Test (COWAT), and Digit Cancellation Test (D-CAT). Values reflect the regression coefficient (β) and standard error (SE) from the adjusted regression that controlled for the same covariates as the primary analyses, along with Nightshift worker status. Statistical significance is denoted by * *p* < 0.05, ** *p* < 0.01, *** *p* < 0.001, with statistically significant associations (*p* < 0.05) bolded.
